# Age distribution and characteristics of choroidal nevi in 35 559 individuals: A nationwide study using optometry fundus photography

**DOI:** 10.1111/aos.70112

**Published:** 2026-03-06

**Authors:** Carsten Faber, Hibba Quhill, Danson Muttuvelu, Jens Folke Kiilgaard

**Affiliations:** ^1^ Department of Ophthalmology Rigshospitalet Copenhagen Denmark; ^2^ Department of Clinical Medicine University of Copenhagen Copenhagen Denmark; ^3^ Ocular Oncology Service Royal Hallamshire Hospital Sheffield UK; ^4^ University of Sheffield Sheffield UK; ^5^ MitØje V/ Danske Speciallæger Aarhus C Denmark

**Keywords:** age distribution, choroidal nevus, MOLES system, population study, prevalence, risk factors

## Abstract

**Purpose:**

To examine the age distribution and characteristics of choroidal nevi in a large nationwide sample.

**Methods:**

We included all individuals who underwent fundus photography in a nationwide chain of optometry stores during a 5‐week period in 2023. Non‐mydriatic 45° or 60° digital fundus images were reviewed asynchronously by a subspecialized ocular oncologist. All images with suspected choroidal nevi, defined as a pigmented choroidal lesion of slate blue or green grey colour, were jointly re‐examined by two ocular oncologists and subsequently reviewed by a third ocular oncologist for confirmation and detailed characterization. Age, sex and geographic distribution were correlated with demographic data from the predominantly white Danish population.

**Results:**

Fundus images from 35 559 individuals aged 5–100 years (median, 54 years; interquartile range, 40–56 years) were included, representing 0.60% of the Danish population. Gradable images of both eyes were available in 31 129 participants, and at least one gradable image was available in 33 588 participants. In total, 852 nevi were identified, corresponding to a crude prevalence of 2.54% (95% CI 2.37%–2.71%). Prevalence increased during the first 5 decades of life (0%, 0.74%, 1.40%, 2.23%, 2.87%, 2.94%, 2.88%, 2.53% and 2.51% in individuals aged <10, 10–20, 20–30, 30–40, 40–50, 50–60, 60–70, 70–80 and >80 years, respectively). Median largest basal diameter of nevi was 1.75 mm (interquartile range 1.33–2.30) and was only weakly associated with age (*R* ^2^ < 0.01; *p* = 0.041). According to the MOLES system, most nevi were classified as common (90.8%) or low‐risk (5.28%), with fewer graded as high‐risk (2.70%) or probable melanoma (1.17%).

**Conclusions:**

The prevalence of choroidal nevi increased throughout the first 5 decades of life. Most nevi were small and lacked risk factors for malignant transformation. This indicates that the emergence of a choroidal nevus can be expected to occur by the age of 50. If supported by longitudinal studies, these findings could inform risk‐based surveillance strategies to optimize resource allocation.

## 1 INTRODUCTION

Choroidal nevi are melanocytic tumours of the choroid. Choroidal nevi differ in certain features such as diameter, presence of orange pigment or serous detachment, which each infer increased risk of developing choroidal melanoma (Gass, 1977, Shields et al., 2019, Stålhammar et al., 2026). These and other risk factors have been incorporated in various mnemonics. Of these, the MOLES system is especially useful for evaluation of choroidal nevi from images alone and allows stratification of a choroidal nevus as either a common nevus, low‐risk nevus, high‐risk nevus or probable melanoma (Damato, 2023). The MOLES system has previously been validated on patients from ocular oncology clinics (Al Harby et al., 2021; Jahnke et al., 2024; Roelofs et al., 2020) and has also been used to describe nevi identified in the Australian National Eye Health Survey (Salehi et al., 2021).

The prevalence of choroidal nevi in the general population is well‐known from large, thorough population studies carried out in India (Nangia et al., 2012), Germany (Elbaz et al., 2019), China (Jonas et al., 2008), Singapore (Ng et al., 2009), the United States (Greenstein et al., 2011; Qiu & Shields, 2015) and Australia (Sumich et al., 1998). The youngest participant in each of these studies was 30, 35, 40, 40, 40, 45 and 49 years old, respectively. A recent study on pooled data from four paediatric population studies has estimated the prevalence of choroidal nevi in children aged 6 months to 18 years of age (Raval et al., 2021). However, an estimate for the prevalence of choroidal nevi in young adults is lacking. Further, the relatively low numbers of choroidal nevi identified in each of the above studies (range 5–287) make it difficult to evaluate the prevalence of subtypes of nevi based on, for example, the MOLES system.

A choroidal nevus is often asymptomatic and the identification in clinical practise is often incidental. With the advent of routine fundus photography and the ease of identification on fundus images, the rate of incidental detection has probably increased in the recent decade. Furthermore, in some countries, optometry chain stores have begun offering fundus photography as part of a standard examination, increasing the pick‐up rate of incidental choroidal nevi (Kern et al., 2020; Muttuvelu et al., 2021). These changes bring a golden opportunity to study choroidal nevi in the optometric cohort of patients and highlight the need for more knowledge on choroidal nevi in the general population including young adults.

Here, the primary purpose was to examine the prevalence and characteristics of choroidal nevi in a large national sample of the general population. A secondary purpose was to examine if this could be carried out using data from an optometry chain.

## 2 METHODS

### 2.1 Study population

The study included consecutive customers of the nationwide optometry chain, Louis Nielsen A/S (Specsavers Denmark), in the 5‐week period of 1 July 2023 to 5 August 2023, who had imaging of their fundus done as part of a routine examination. The photos with data on age and sex were uploaded to a cloud‐service allowing for asynchronous evaluation. Geographical location of the store was used as a proxy measure for regional residence of each individual. Data were handled in a pseudo‐anonymous matter allowing for identification of multiple examinations of the same individual. Storage and management of data complied with the General Data Protection Regulation. This telemedicine setup has been described in detail elsewhere (Muttuvelu et al., 2021).

### 2.2 Imaging and grading

All fundus photographs were acquired non‐mydriatically using a 45° colour digital camera (iCare DRS, Finland or Topcon Maestro2, Denmark) or a 60° scanning laser camera (iCare DRSplus, Finland). Images were centred on the fovea. Where available, red‐free and infrared images were included.

All images were assessed by an ocular oncologist (C.F. or J.F.K.) for presence of nevi. A nevus was defined according to previous population studies as a pigmented choroidal lesion of slate blue or green grey colour. To allow for follow‐up studies, a minimal diameter was not used. Images from individuals who were identified with a nevus in the primary assessment were graded jointly (C.F. and J.F.K.) and then submitted for regrading by a third ocular oncologist (H.Q.). This involved MOLES grading that was done blinded to the primary grading.

### 2.3 Statistics

We used demographical data on the predominantly white Danish population on 1 July 2023 available from Danish Statistics (www.dst.dk/en). Prevalence estimates and 95% confidence intervals were calculated using the Wilson score method. The association between age and largest basal diameter (LBD) was estimated using linear regression. Linearity was assessed using a LOESS curve. Inter‐grader agreement for MOLES score was assessed using weighted Cohen's kappa. All analyses were performed using R Statistical Software (v4.4.3; R Core Team 2025).

### 2.4 Ethics

Fundus‐photography is part of a standard examination in Louis Nielsen A/S (Specsavers Denmark). The customers were informed and consented to this, including the broad use of data for internal quality and research. Our study follows the Tenets of the Declaration of Helsinki and has not led to additional imaging or other interventions. Ethics approval for this study was waived by the Regional Ethics Committee (F‐22041899). All incidental findings identified during this study that warranted referral (e.g., retinal detachment, exudative age‐related macular degeneration, high‐risk nevi or suspected melanomas) were reported to the headquarters of Louis Nielsen A/S (Specsavers Denmark) to ensure that each patient had been, or would be, appropriately referred for further examination.

## 3 RESULTS

### 3.1 Population

**FIGURE 1:**
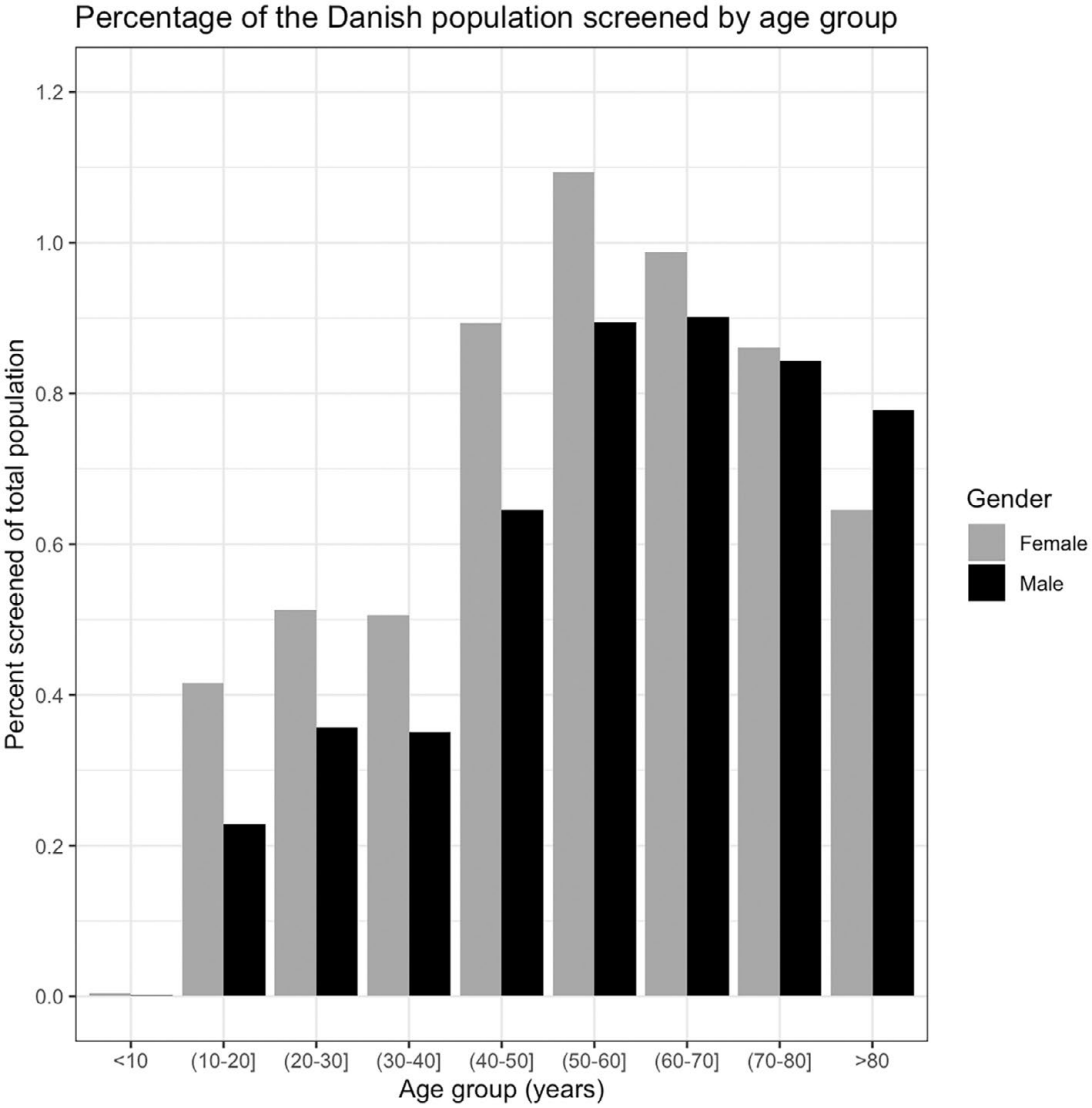
Individuals included in study as per cent of total population in Denmark.

We included 35 559 unique individuals (median age: 54 years; interquartile range 40–66). This corresponded to 0.60% of the Danish population (as of 1 July 2023) and represented individuals from all five regions in Denmark (Capital Region 0.37%, Region Zealand 0.68%, Region of Southern Denmark 0.57%, Central Denmark Region 0.56%, North Denmark Region 0.63%). The proportion of the Danish population included in the study varied by the age group: for those aged 10–20 years, 0.23% and 0.42% of the male and female Danish population were included. In the age group 50–60 years, 0.89% and 1.09% of the male and female Danish population were included (Figure 1).

Of the included individuals, 19 929 (56%) were female, 31 127 (88%) had gradable images of both right and left eyes, 2459 (6.9%) had a gradable image of one eye only and 1973 (5.5%) had zero gradable images (Table 1). The proportion of gradable images varied by patient age and was highest in those aged 20–30 years (Figure S1). Most were imaged with 45° colour fundus cameras (DRS, 91.2%; Maestro 2, 5.56%), while a minority were imaged with a 60° scanning laser camera (DRSplus, 3.25%).

**TABLE 1:** Characteristics of included individuals.

Characteristic	*n* = 35 559^a^
Age (years)	54 (40, 66)
Age groups	
(−Inf, 10]	20 (<0.1%)
(10, 20]	2189 (6.2%)
(20, 30]	3440 (9.7%)
(30, 40]	3136 (8.8%)
(40, 50]	5655 (16%)
(50, 60]	8042 (23%)
(60, 70]	6368 (18%)
(70, 80]	4835 (14%)
(80, Inf]	1874 (5.3%)
Gender	
Female	19 929 (56%)
Male	15 630 (44%)
No. of gradable images	
0	1973 (5.5%)
1	2459 (6.9%)
2	31 127 (88%)

^a^ Median (Q1, Q3); *n* (%).

### 3.2 Prevalence and age distribution of nevi

**FIGURE 2:**
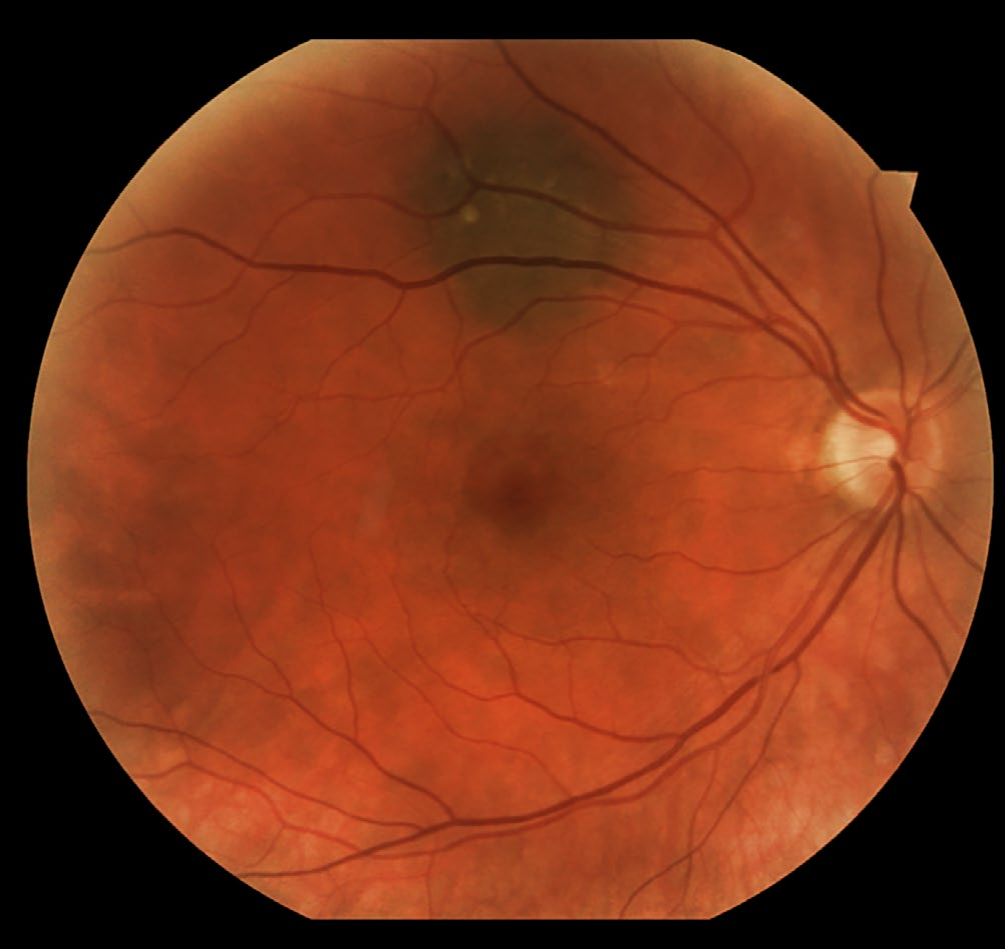
Non‐mydriatic 45° fundus photograph included in the study, showing a choroidal nevus with drusen and a MOLES score of 0.

We identified one or more nevi in 852 individuals out of 33 586 individuals with at least one gradable image corresponding to a crude prevalence of 2.54% (95% CI 2.37%–2.71%). A representative example is shown in Figure 2. Nevi were identified in 443 females and 409 males corresponding to a gender‐specific prevalence of 2.33% (95% CI 2.13%–2.56%) in females and 2.80% (95% CI 2.55%–3.08%) in males.

Of the 852 individuals, nevi were identified in the right eye (432 cases, 50.7%), left eye (402 cases, 47.1%) or in both eyes (18 cases, 2.11%). Thirty‐seven individuals (4.34%) had two nevi in one eye, one individual had three nevi in one eye (0.1%).

**FIGURE 3:**
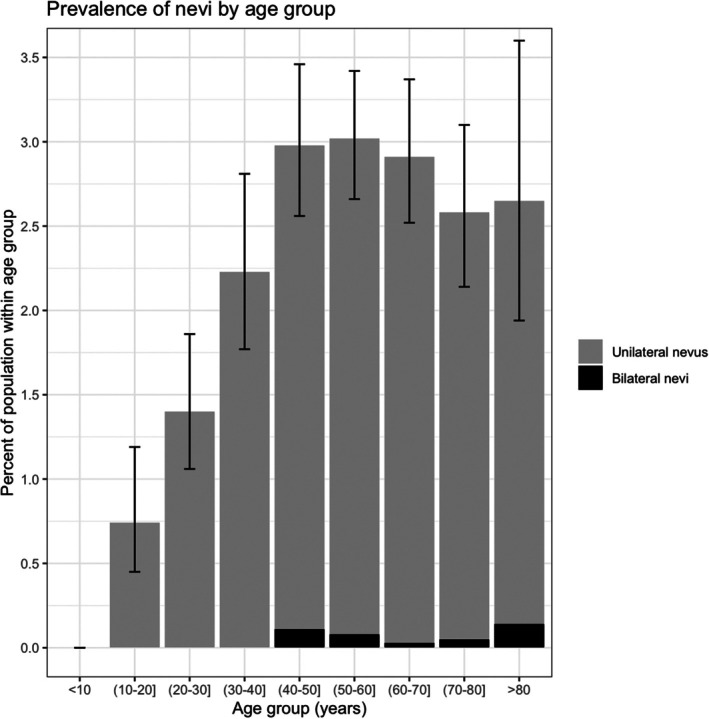
Prevalence of choroidal nevi by age group as per cent of total population in Denmark. Error bars indicate 95% confidence intervals.

The prevalence of nevi increased during the first 5 decades of age (0%, 0.74%, 1.40%, 2.23%, 2.87%, 2.94%, 2.88%, 2.53% and 2.51% in individuals aged <10, 10–20, 20–30, 30–40, 40–50, 50–60, 60–70, 70–80 and above 80 years, respectively; Figure 3).

The youngest individual with two or more nevi in one eye was 30.0 years old. The youngest individual with nevi in both eyes was 42.6 years old.

### 3.3 Characteristics of nevi

Of the 852 individuals with nevi, 774 (90.8%) had a MOLES score of 0, 45 (5.28%) had a MOLES score of 1, 23 (2.7%) had a MOLES score of 2 and 10 (1.17%) had a MOLES score of 3 or higher. The MOLES score was primarily attributed to the prevalence of trace or confluent orange pigment (66 cases, 7.74%) and a LBD above 4.5 or 6 mm (23 cases, 2.70%).

**FIGURE 4:**
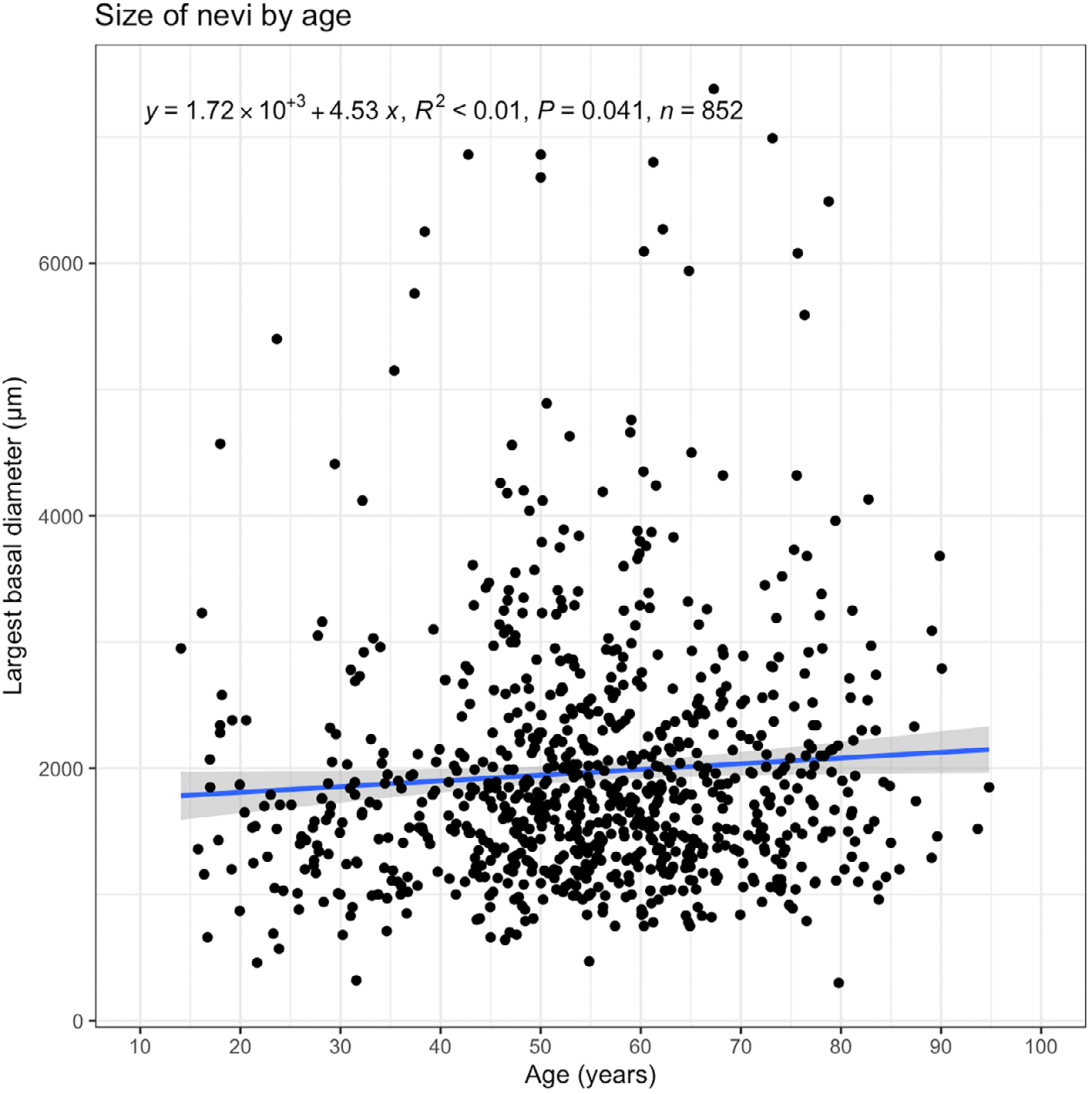
Size (largest basal diameter) of all nevi by age with linear regression.

The median LBD was 1.75 mm (interquartile range 1.33–2.30). While we observed an increased diameter with age, this association was weak (4.53 μm/year, *R* ^2^ < 0.01 *p* = 0.041, Figure 4). No substantial deviations from linearity were observed when comparing the LOESS curve to the fitted linear regression line. The exact LBD could not be determined in 114 cases (13.4%) due to partial visualization of the nevus at the edge of the image.

### 3.4 External validation

All images that were identified with a nevus in the primary examination were re‐examined by an external grader (H.Q.). This re‐examination confirmed the presence of at least one nevus in all 852 cases. Regrading based on the MOLES classification was conducted in a blinded manner. The crude percentage agreement was 92.3%. Quadratic weighted Cohen's kappa indicated moderate to substantial inter‐grader agreement (*κ* = 0.60). A difference was noted in the MOLES score of 1 point in 46 (5.4%) cases, and 2 points in 20 cases (2.3%).

## 4 DISCUSSION

Using optometry store fundus photography obtained during routine examination, we examined a large proportion of the Danish population and found that choroidal nevi were relatively common and that most were without risk‐factors for malignant transformation. We observed a higher prevalence of nevi in older individuals, suggesting possible age‐related patterns in detection or development.

We found a crude prevalence of choroidal nevi of 2.54% (95% CI 2.37%–2.71%), which is similar to the crude prevalence of 2.5% in a recent study from Gutenberg in Germany (Elbaz et al., 2019). A higher prevalence has been noted in other studies (Gordon‐Shaag et al., 2014; Greenstein et al., 2011; Qiu & Shields, 2015; Sumich et al., 1998), which may be explained by a higher age of included individuals and the imaging of a larger proportion of the posterior fundus. The topographical distribution has previously been examined. Based on OPTOS 200° imaging, it has been found that 61% of nevi in young adults are present within the posterior 70° (Gordon‐Shaag et al., 2014) and that two‐thirds of nevi in adults are present within the posterior 60° (Yeşiltaş et al., 2024). A clinical based study reported similarly that most choroidal nevi were present in the centre of the posterior pole (Krohn et al., 2009).

The increased prevalence of choroidal nevi during the first 5 decades of life has not, to the best of our knowledge, been documented before in a large study. Gass described a similar finding in a clinic‐based case‐series on 250 individuals of which 23 were below 40 years of age (Gass, 1977). It has also been implied in a recent review on prevalence of choroidal nevi (Yeşiltaş et al., 2024), which included studies of individuals aged below 18 and above 45 years of age. The study from Gutenberg included individuals from 35 years of age and older, but did not include data on distribution of choroidal nevi within age‐groups (Elbaz et al., 2019).

While the melanocytes forming a nevus are probably present in the choroid from birth, our finding of increased prevalence with age suggests that melanocytic hyperpigmentation which allows identification takes place at a later stage. From this it can be implied that the emergence of a choroidal nevus is an inherent characteristic and not necessarily a marker for malignant transformation. This implication challenges the MOLES system where the emergence of a small nevus results in the grading as a high‐risk nevus (Damato, 2023).

We found that the prevalence of choroidal nevi decreased in the higher age‐groups. This may reflect an underestimation due to imaging issues such as more opaque media (e.g. cataracts) and miotic pupils with age. Similar findings have also been reported by others (Greenstein et al., 2011; Sumich et al., 1998). In Gutenberg Health Study, the largest population study carried out on choroidal nevi, an association between prevalence of choroidal nevi and previous myocardial infarction was reported offering another explanation (Elbaz et al., 2019).

We found no cases of bilateral nevi before the age of 30 years and a prevalence of bilateral nevi between 0.03% and 0.14% for individuals aged 40 or older. This agrees with reports of no bilateral nevi in children below 20 years of age (Yeşiltaş et al., 2024) and a prevalence of bilateral nevi at 0.06% in individuals aged 40 or older (Ng et al., 2009).

The majority of nevi were common (90.8%) or low‐risk nevi (5.28%). This is comparable to a recent Australian population study where 97% of 77 nevi were graded as common nevi (Salehi et al., 2021). In our study, the median LBD of nevi was 1.75 mm and we estimated a growth rate of 4.53 μm/year. However, because this is a cross‐sectional study, we cannot make such causal inferences. Nonetheless, this growth rate of nevi corresponds to the reported growth rate of 6 μm/year of nevi in the Blue Mountains Eye Study (Thiagalingam et al., 2004). This compares to higher rates observed in larger lesions, such as a collection of larger nevi (median diameter 5 mm, median height 1.5 mm) with a growth rate of 60 μm/year (Mashayekhi et al., 2011) and an even higher growth rate of 250 μm/year reported for presumed incipient choroidal melanoma (Jouhi et al., 2023).

The nationwide inclusion of individuals during a short period of time is a strength of our study. Compared to previous large population studies, we included a very large number of individuals with a relatively high proportion aged below 40 years of age. However, there are limitations: The cross‐sectional design prevents conclusions about causality between age and the prevalence or size of nevi. Further, reliance on non‐mydriatic 45° fundus photography may have underestimated prevalence due to limited fundus coverage and reduced image quality, particularly in older individuals. The proportion of non‐gradable images was, however, comparable to other studies (Elbaz et al., 2019; Nangia et al., 2012). Our study also relied solely on colour fundus photography without the benefit of imaging modalities such as autofluorescence, optical coherence tomography and ultrasonography to identify and characterize choroidal nevi (Cennamo et al., 2018). This is mitigated somewhat by the MOLES system having been devised to be used with fundus photography alone (Damato, 2023), unlike the TFSOM‐DIM system (Damato, 2023; Shields et al., 2019).

We found that choroidal nevi were relatively common in the Danish general population and that most of these were without risk‐factors for transformation into melanoma. Even though Denmark has one of the highest incidences of choroidal melanoma world‐wide (Wu et al., 2023), choroidal melanoma is still a rare disease. Nonetheless, identifying choroidal melanoma at an early stage increases survival and results in better chances of vision‐saving and globe‐saving treatments (Ah‐Fat & Damato, 1998; Shields et al., 2009; Smidt‐Nielsen et al., 2021). Together, this implies that routine annual surveillance of all nevi may not be necessary. We suggest an individualized surveillance scheme would be more appropriate. If validated through longitudinal studies, this should be based on nevus risk‐factors and incorporating age‐specific prevalence information to identify those nevi at highest risk of transforming into melanoma at an early stage.

In conclusion, this study identified a prevalence of 2.5% for choroidal nevi in the Danish population based on fundus photography obtained from optometry stores. The majority of nevi were small and exhibited no risk factors for malignant transformation into choroidal melanoma. Prevalence was observed to increase during the first 5 decades of life. Longitudinal follow‐up studies are warranted to validate these findings and assess long‐term risk.

## Supporting information


Figure S1.
