# Supplementary material for: Age distribution and characteristics of choroidal nevi in 35 559 individuals: A nationwide study using optometry fundus photography
Source: Acta Ophthalmol. 2026 Mar 6;104(6):e655–61. doi: 10.1111/aos.70112 (PMC13460363; doi:10.1111/aos.70112)
Supplement: Supplementary file 1 [file AOS-104-e655-s001.pdf]

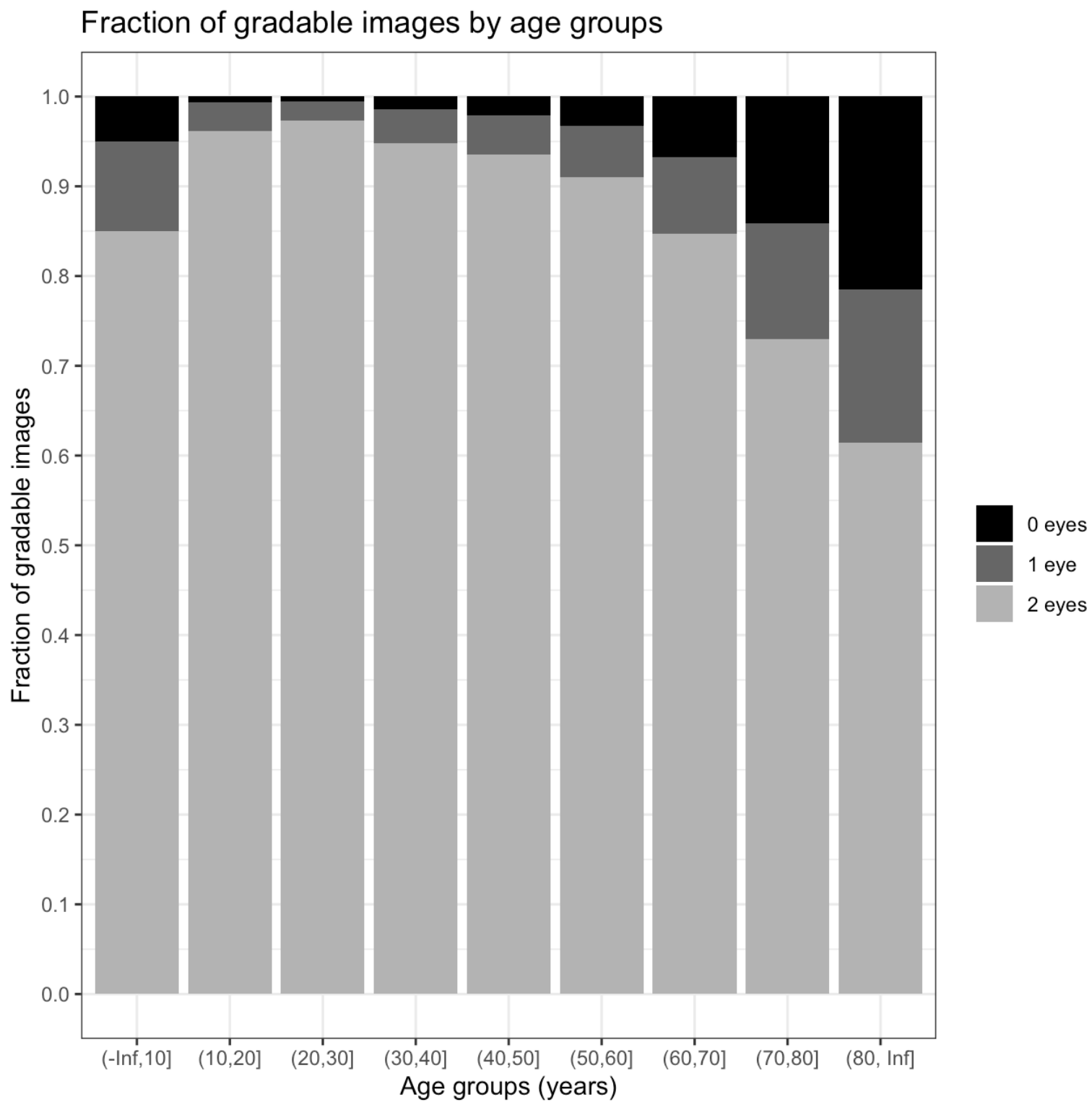

Supplemental figure 1. Fraction of gradable images by age groups
